# Multipotent Stromal Cells and Viral Interaction: Current Implications for Therapy

**DOI:** 10.1007/s12015-021-10224-9

**Published:** 2021-08-04

**Authors:** Nopmanee Taechangam, Amir Kol, Boaz Arzi, Dori L. Borjesson

**Affiliations:** 1 Department of Pathology, Microbiology and Immunology, School of Veterinary Medicine, University of California, Davis, Davis, CA USA; 2 Department of Surgical and Radiological Sciences, School of Veterinary Medicine, University of California, Davis, Davis, CA USA

**Keywords:** Multipotent stromal cells, Mesenchymal stem cell, Virus interaction, Viral disease, Antiviral immunity

## Abstract

Multipotent stromal cells (MSCs) are widely utilized in therapy for their immunomodulatory properties, but their usage in infectious viral diseases is less explored. This review aimed to collate the current novel use of MSCs in virus-associated conditions, including MSC’s susceptibility to virus infection, antiviral properties of MSCs and their effects on cell-based immune response and implementation of MSC therapy in animal models and human clinical trials of viral diseases. Recent discoveries shed lights on MSC’s capability in suppressing viral replication and augmenting clearance through enhancement of antiviral immunity. MSC therapy may maintain a crucial balance between aiding pathogen clearance and suppressing hyperactive immune response.

**Graphical Abstract:**

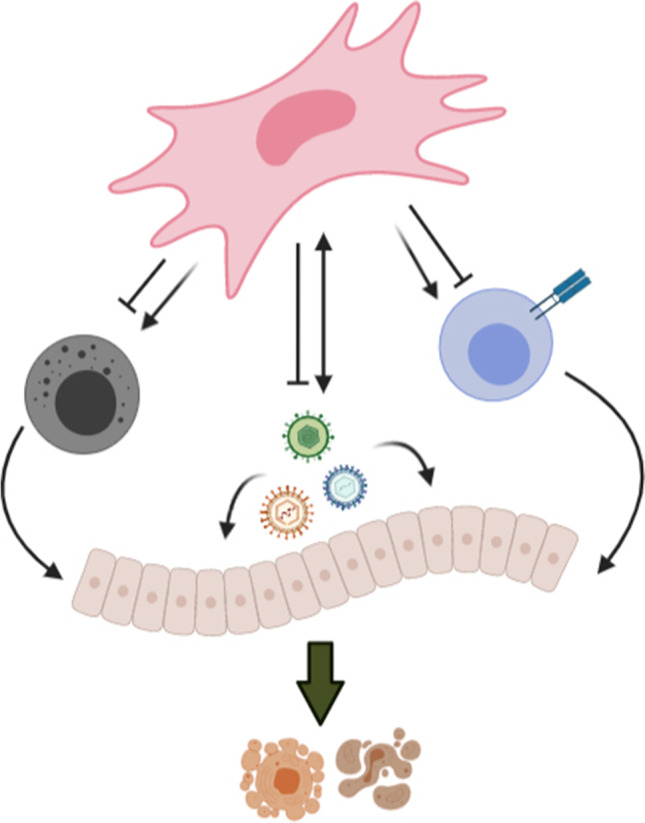

## Introduction

Multipotent stromal cells (MSCs) can be isolated from a variety of tissues, expanded ex-vivo, and administered to patients as a therapeutic agent. MSCs undergo differentiation into mesenchymal tissue types and secrete trophic factors to aid in the regeneration and repair of damaged tissue [1, 2]. MSCs further interact with various types of immune cells and their potent immunomodulatory properties are being investigated in numerous clinical trials [3].

MSCs inhibit NK cell and T-cell proliferation [4, 5], induce Treg differentiation [6], reduce the differentiation of B-cells to antibody-secreting plasma cells [7], and shift monocytes and dendritic cells to a regulatory phenotype [8, 9]. However, MSCs may exert differential effects depending on the local microenvironment [10], adding even more to the complexity of understanding MSC-mediated immunomodulation. MSC express pattern recognition receptors (PRR) such as Toll-like receptors (TLR), retinoic acid-inducible gene I-like receptors (RLRs) and nucleotide binding domain-like receptors (NLRs). Ligation of PRRs leads to downstream MSC cell signaling cascades and cell activation [11].

While MSC’s immunomodulatory effects with a shift towards peripheral tolerance are very well studied, [12, 13] MSCs are also capable of augmenting anti-bacterial responses and can produce antimicrobial peptides, enhanced by the presence of bacteria [11, 14, 15]. While MSCs contribute to host defense and inflammation, there are limited data on MSC use in infectious diseases, particularly in viral infections.

The objectives of this review are 1) to collate current data on virus-MSC interactions and MSC interaction with cells that are at the forefront of anti-viral immunity, 2) to discuss the current status of MSC therapy in the context of viral diseases, including COVID-19, 3) to review animal models of viral disease and on-going human MSC clinical trials, and 4) highlight key gaps in knowledge and future research opportunities.

## Viral Infection of MSCs, Potential Outcomes and Safety

MSCs are susceptible to infection by a wide variety of RNA and DNA viruses both in vitro and in vivo [16–23]. MSCs possess numerous functional surface receptors [24], which potentially could facilitate viral entry. Although virus receptors vary in structure and function, they are more inclined to utilize molecules involved in cellular adhesion [25], for example, I-CAM1 which MSCs express and use for transmigration and immunomodulation [26].

MSC surface receptor expression and viral tropism may partially explain MSC susceptibility to viral infection. MSCs are highly permissive to infection by many genera of Herpesviruses, including Herpes Simplex-1 (HSV-1), Varicella Zoster virus (VZV) and Cytomegalovirus (CMV) [27]. HSV-1 can infect MSCs through the heparan sulfate receptor [28]. However, Epstein-Barr virus (EBV) and Human Herpesvirus-6, 7 and 8 (HHV) were unable to infect human MSCs despite MSC expression of receptors known to facilitate viral entry into other cells [29, 30]. This may be explained by cellular tropism as EBV typically resides in B-cells and HHV typically infects T-cells while HSV-1, VZV and CMV primarily infect epithelial cells at various sites of mucosal membranes [31].

Various factors play a role to determine viral tropism, including surface binding receptors on target cells, antiviral signaling of cytokines, availability of intracellular host factors which supports viral RNA/DNA synthesis and activation state of the cell [32].

Although MSCs clearly are susceptible to viral infection, MSCs possess some resistance not observed in other somatic cells, partially due to their intrinsic upregulation of interferon-stimulated genes (ISGs) [33]. Stem cell pluripotency has been correlated to their resistance to viral infection and the resistance to viral infection is more robust in embryonic stem cells (ESCs) and induced pluripotent stem cells (iPSCs) and less so in highly differentiated cells, as shown an in vitro study where MSC-derived cells were permissive to HIV-1 infection, but that same virus could not productively infect undifferentiated MSCs [34].

**Fig. 1:**
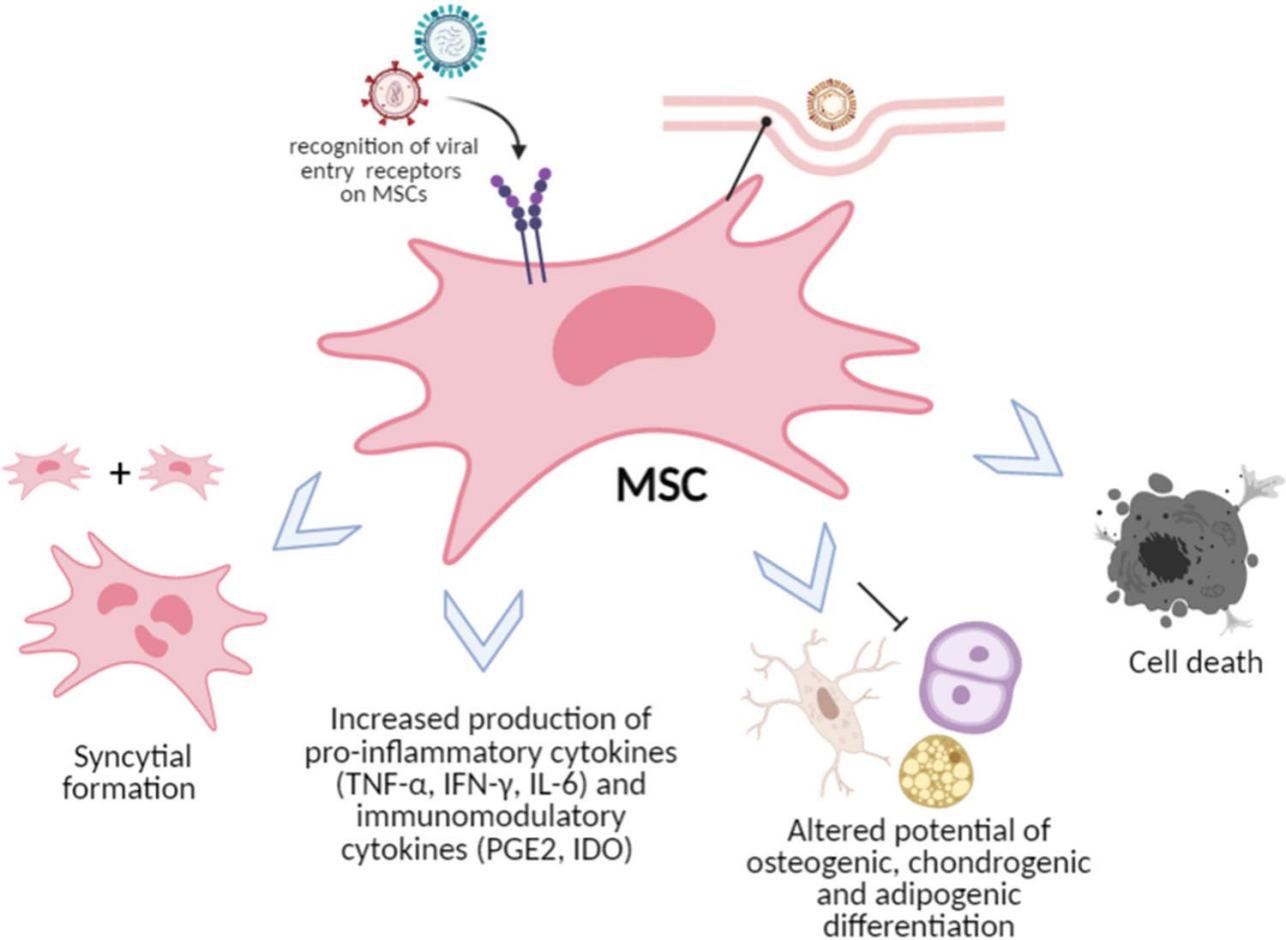
Possible outcomes of viral infection on mesenchymal stem cells. TNF-α: tumor necrosis factor-alpha; IFN-γ: interferon-gamma; IL-6: interleukin-6; PGE2: prostaglandin E2; IDO: indoleamine-2,3-dioxygenase

Outcomes of viral entry into MSCs are variable and may result in either MSC death, persistent infection or cellular transformation which impairs their functionality. In general, RNA viruses are more effective in initiating pro-inflammatory cytokine production and eliciting an immediate antiviral response from MSCs (Fig. 1 and Table 1). This is in line with the strategy that infection with the majority of RNA viruses, with the exception of retroviruses, is more likely to cause an acute infection rather than chronic conditions, compared to DNA viruses [35]. Additionally, RNA viruses have more disordered viral protein packaging and a small genome size. These factors may advantage the prompt initiation of conformational changes necessary during host-cell entry and interaction [36]. For example, Chikungunya virus (CHIKV) can dampen MSC’s osteogenic differentiation, potentially limiting their use in regenerative medicine [37]. However, the ability to elicit an inflammatory reaction is not solely limited to RNA viruses. Parvovirus B19, a single-stranded DNA virus, can infect human BM-MSCs with resultant upregulation of pro-inflammatory cytokine gene expression, such as, IL-6 and TNF-α [17].

**Table 1:** Viral susceptibility of MSCs

Family	Virus	Type	MSC sources and species	Outcome of Infection
Pneumoviridae	Respiratory syncytial virus (RSV) [19]	ssRNA	Human BM-MSCs	Increased expression of IFN-β and IDO, enhanced MSC’s capability of PBMC inhibition of proliferation
Orthomyxoviridae	Human and Swine Influenza virus (H1N1) [21]	ssRNA	Human/Porcine BM-MSCs	Increased production of pro-inflammatory cytokines (TNF-α and IL-6), CPE
	Avian Influenza virus (H1N1 and H9N5) [38] (H5N1)		Chicken pulmonary MSCs Human UC-MSCs and BM-MSCs	Increased production of cytokines (IL-6 and IL-8), CPE
Retroviridae	Human Immunodeficiency Virus (HIV) [39, 40] Feline Foamy Virus (FFV) [18] Simian Foamy Virus (SFV) [41]	ssRNA	Human BM-MSCs Feline AD-MSCs Rhesus Monkey BM-MSCs	Increased adipogenic potential, Impaired osteogenic differentiation, Induced senescence Syncytial formation, Impaired proliferation, CPE
Togaviridae	Chikungunya virus (CHIKV) [37]	ssRNA	Human BM-MSCs	Impaired osteogenic differentiation
Birnaviridae	Infectious bursal disease virus (IBDV) [42]	dsRNA	Chicken BM-MSCs	CPE
Herpesviridae	Cytomegalovirus (CMV), Herpes simplex virus type 1 (HSV-1) [29] Varicella zoster virus (VZV), HSV6-8, [16] Kaposi sarcoma-associated herpesvirus [43]	dsDNA	Human BM-MSCs, Human fetal MSCs	CPE Impaired immunosuppressive function (CMV)
Hepadnaviridae	Hepatitis B virus (HBV) [22]	dsDNA	Human BM-MSCs	Maintained MSCs characteristics
Parvoviridae	Parvovirus B19 virus [17, 44]	ssDNA	Human synovial and BM-MSCs	Maintained MSCs characteristics [44] Increased expression of IL-6 and TNF-α [17]

CPE: cytopathic effects (MSC lysis), BM-MSCs: bone marrow derived MSCs,

UC-MSCs: umbilical cord derived MSCs

To illustrate the consequences of DNA virus infection, MSCs infected with CMV lost their cytokine-induced immunomodulatory function and were no longer capable of inhibiting microbial growth [23]. In addition, the US11 protein utilized by CMV for immune evasion can also downregulate MHC class I expression on human MSCs, making them vulnerable to NK cell-mediated lysis [45]. This same effect was described in horse MSCs after equid herpesvirus-1 (EHV-1) infection [20].

The ability of viruses to enter and alter host MSCs may also be host species dependent. Human and murine MSCs secrete different immunomodulatory mediators [46] and the efficacy of these mediators to limit or enhance viral replication may be an important determinant of infection outcome. For example, indoleamine-2,3-dioxygenase (IDO) is a primary mediator utilized to mitigate viral replication in human MSCs, but the same effect was not observed in murine MSCs [47].

Due to the concern over the ability of viruses to infect MSCs, tissues from candidate donors are screened for common viral infection and expanded allogeneic MSC doses are also screened for the presence of viral infection prior to cell administration. Although allogeneic MSCs may potentially serve as a reservoir for latent viruses, especially if administered to immunocompromised recipients, clinical studies in GvHD patients have shown that MSC treatment did not induce more viral reactivation as compared with conventional immunosuppressive therapy [48]. Overall, current data suggests that MSC therapy is deemed largely safe for with minimal virus-associated risk.

## Anti-Viral Properties of MSCs

Despite their permissiveness to some viral entry, evidence has also emerged that MSCs can mitigate viral infection via upregulation of their antiviral mechanisms. MSCs are more resistant to viral infections when compared to more differentiated cells through their intrinsic upregulation of IFN-stimulated genes (ISG) which block viral replication and propagation [33]. Moreover, silencing ISG such as p21/CDKN1A and IFITM3 expression in MSCs, resulted in increased susceptibility of MSCs to chikungunya virus infection and zika virus respectively [33, 49].

Several in vitro studies have demonstrated antiviral activity of MSCs, for example, MSCs can inhibit inflammasome activation in the presence of Coxsackievirus B3 [50] Another described antiviral mechanism of MSCs is through their non-coding miRNAs. Some MSC’s released miRNAs demonstrated vigorous antiviral activity that could inhibit Hepatitis C virus infection [51]. With these limited data, primary mechanism of extracellular viral inhibition may possible be from secreted trophic factors.

In vivo murine studies of influenza virus-induced acute lung injury have demonstrated that MSC administration reduced pulmonary injury and inflammation and restored alveolar fluid clearance [50, 52]. In a mouse model of murine gammaherpesvirus-68 (MHV-68) infection, MSCs also showed anti-herpesviral properties, mediated by a cytosolic DNA sensing pathway, and MSCs also limited intracellular viral replication in IFN-γ dependent and independent manners [53]. However, the ability of MSCs to inhibit viral replication in vivo and the mechanisms involved require further investigation.

## Effects of MSCs on the Cell-Based Immune Response Against Viral Infection

**Fig. 2:**
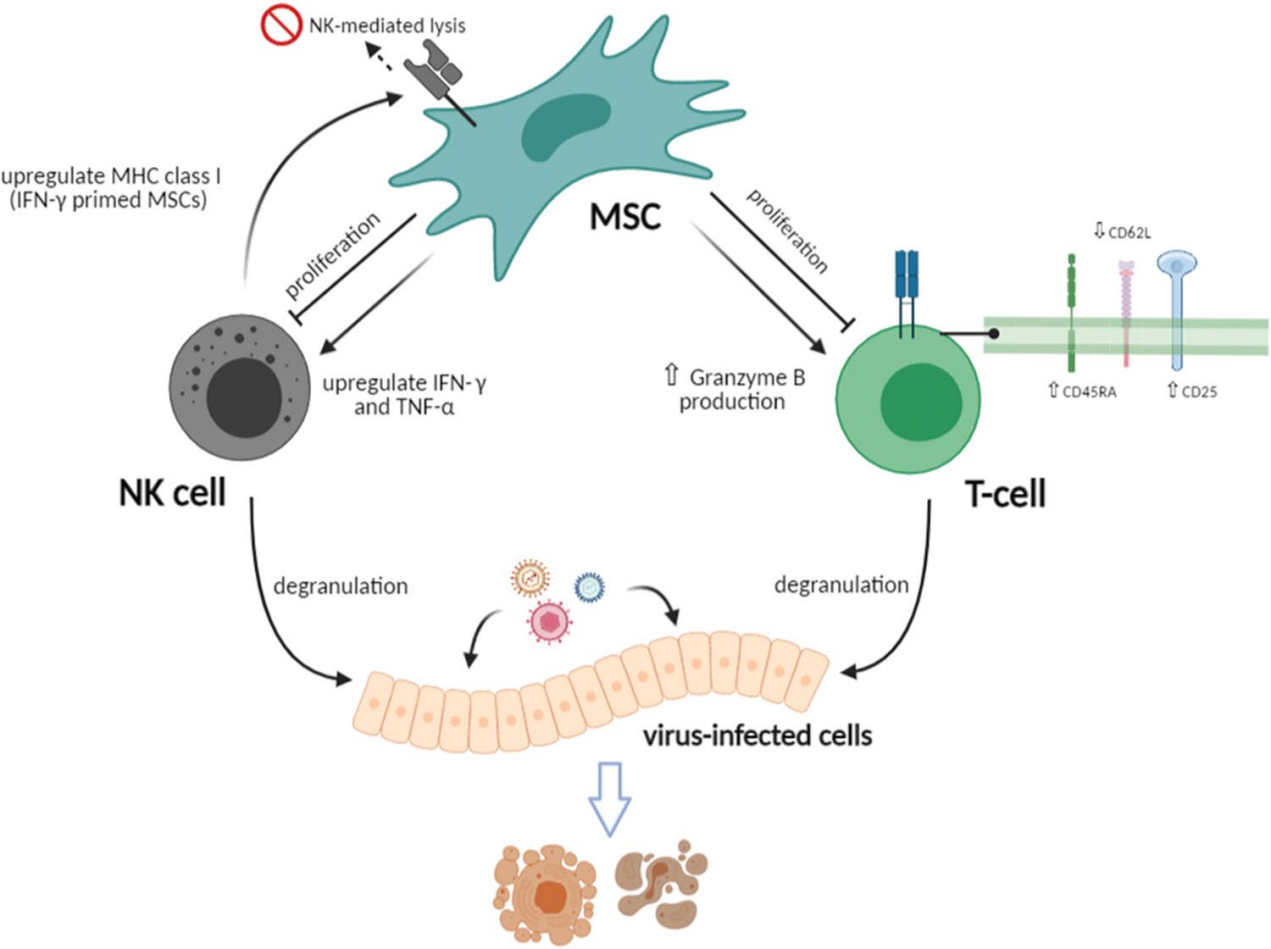
MSCs-induced alterations on NK cells and T-cells. TNF-α: tumor necrosis factor-alpha; IFN-γ: interferon-gamma

Aside from their intrinsic restriction factors, MSCs can also modify the antiviral response of the immune cells normally implicated in antiviral defenses. MSCs can interact with and influence both the innate and adaptive immune cellular components, primarily on NK cells and T-cells, potentially altering the outcome of the response to viral infection (Fig. 2).

Despite their intensive studies, the data on MSC interactions with NK and CD8 + T-cells are conflicting and complex. Autologous and allogeneic cultured MSCs can be recognized and killed by activated NK cells. However, IFN-γ primed MSCs, mimicking exposure to inflammatory environment, upregulate MHC class I expression and avoid NK-cell mediated destruction [54]. While they can be targets, MSCs can also influence and alter NK cells’ phenotype. Human MSCs inhibited NK cell proliferation, decreased cytokine production and dampened cell differentiation to fully functional effector cells in vitro. These effects were mediated by the soluble mediators IDO and prostaglandin E2 (PGE2) and resulted in the downregulation of NK cell surface receptors [55]. A more recent study demonstrated that MSCs upregulated NK cell secretion of IFN-γ and TNF-α and also triggered their degranulation, increasing the release of perforin and granzyme and enhancing NK cell’s effector phenotype [56]. The discrepancies may be due to variations in study designs. The first study examined pre-activated human NK cells that had been cultured with IL-2 for 7 days while the latter study used short-term activated human NK cells cultured with a more diverse combination of cytokines. Furthermore, the ratios of NK cells to MSCs in experimental settings also play a role in demonstrated level of suppression [57].

With CD8 + T-cells, it is well established that MSCs can inhibit T-cell proliferation [58, 59] through the release of transforming growth factor beta (TGF-β) and hepatocyte growth factor (HGF), which leads to the decrease of cyclin D2, causing proliferation arrest in the G0G-1 phase of cell cycle [60]. However, MSCs do not appear to hinder CD8 + T-cell cytotoxicity function. After exposure to an exogenous peptide, CD8 + T cells retained the ability to lyse target cells even in the presence of human MSCs [61]. Murine MSCs enhanced granzyme B production and induced degranulation of activated CD8 + T-cells in vitro [62] yet this upregulating effect was not demonstrated in human MSCs [63]. MSCs may reduce cytotoxicity-mediated lysis, only when the T-cells had not been pre-activated (naïve) [64] which may not emulate realistic clinical conditions where MSC therapy would be administered and expected to exert their effects on antigen-experienced CD8 + T-cells.

Additionally, MSCs did not affect the expansion and function of virus-specific CD8 + T-cell in the context of EBV and CMV infection. However MSCs were capable of suppressing alloreactive T-cells [65]. A separate study showed that MSCs may inhibit proliferation of virus-specific CD8 + T-cells, but the experiment was performed by briefly pulsing T-cells with CMV phosphoprotein and Influenza matrix protein antigen for 2 h [66]. This activation may result in a less robust outcome compared to the generation of virus-specific T-cells for 14 days to mimic actual viral exposure. IFN-γ derived from MSCs was hypothesized to play a role in offsetting the immunosuppressive effect of MSCs by mediating the partial cytotoxic responses during viral infection [67].

In addition to affecting T-cell proliferation, MSCs can also alter the activation and differentiation process of T cells. MSCs are known to promote generation of regulatory T-cells [68]. A recent study shed light that this promotion of regulatory T-cells from MSCs arises from an epigenetic conversion of conventional T cells to regulatory phenotype rather than expansion of natural regulatory T-cells [69], increased population of regulatory T-cells have been shown to improve influenza virus clearance in a murine study [70].

The ability of MSCs to alter immune cell functions is likely dependent on host species and varying inflammatory conditions. Several contradictory findings showed that MSCs were unable to suppress or even enhance T cell responses under several conditions. Since MSCs respond differentially to the dynamic changes of inflammatory factors, the immunoregulation of MSCs is distinctly plastic [10] and does not occur intrinsically, but activated by certain combinations of inflammatory cytokines, IFN-γ with TNFα, or IL-1β [71]. Therefore, MSCs’ impact on subsets of virus-associated immune cells will differ under various pathological settings.

## MSC Therapy in Animal Models of Viral Diseases

MSCs and/or their secretome have been used as a therapy in several animal models of viral diseases. These studies have demonstrated that MSCs can reduce inflammation and dampen pro-inflammatory cytokine production, but also demonstrated more novel outcomes including targeting viral-sequestered cells and tissues and directly inhibiting viral expression and replication (Table 2).

**Table 2:** Multipotent stromal cells and their exosome-derived components in the treatment of viral diseases

Animal Models				
Target Virus	Treatment	Species	Viral Kinetics	Outcome
Japanese Encephalitis Virus (JEV)	BM-MSCs	Mice	Decreased viral load in brain tissue and reduced viral propagation	Reduced mortality, alleviated inflammatory response (neuronal damage and blood brain barrier destruction)
Coxsackievirus B3 (CVB3)	BM-MSCs	Mice	Reduced intracellular viral particle production and viral progeny release in cardiomyocytes	Improved CVB3-induced myocarditis, mitigated cardiac apoptosis, cardiomyocyte damage and cardiac mononuclear cell activity
Hepatitis B Virus	BM-MSCs	Mice	Enhanced viral gene expression and replication in vivo	Attenuated immune-mediated liver injury, reduced pro-inflammatory cytokine production cellular response
Swine Influenza Virus	MSCs derived exosomal microRNAs	Pig	Inhibited viral replication lung tissue and reduced viral shedding in nasal swabs	Alleviated SwIV-induced acute lung injury and decreased production of pro-inflammatory cytokines
Simian Immunodeficiency Virus	Allogeneic BM-MSC	Rhesus macaque	Decreased peripheral viral loads	Clearance of virus from gut effector sites, robust regeneration of germinal centers and restoration of follicular T helper cells
*In vitro *experiments				
Hepatitis C Virus	Umbilical MSCs derived exosomes	Human	Inhibition of viral infection through paracrine signaling with synergistic effect upon combination with IFN. No effects on HCV entry into target cells	
Human Immunodeficiency Virus (HIV)	AD-MSCs	Human	Enable potent HIV reactivation in latently infected monocytic and T-cell lines through PI3K-NFκB signaling pathway	

The murine model has been used to study Japanese encephalitis virus (JEV), Hepatitis B and Coxsackievirus B3 virus (CVB). Bone marrow (BM)-derived MSCs have been used to treat all 3 viral infections in the mouse model. JEV is the leading cause of viral encephalitis in Asia and the mouse has disease manifestations that mimic the symptoms and biomarkers observed in humans [72]. The administration of intravenous murine BM-MSCs to mice infected with JEV resulted in a direct antiviral effect both in vitro and in vivo as evidenced by a reduction of viral load in cerebral tissue, decreased inflammatory response and neuronal damage and a reduction of viral propagation in Neuro2a cells in co-culture with MSCs [73]. This antiviral effect of murine BM-MSCs was found to be mediated by the induction of IFN-α and β expression in infected cells. The JEV study by Bian et. al is one of the first animal studies to demonstrate a novel concept that MSCs can directly hinder viral replication in vivo.

CVB infection in the mouse results in myocarditis initiated both by immune-mediated mechanisms and by direct viral-induced cardiomyocyte injury. Similar to JEV, the administration of MSCs reduced intracellular viral particle production and viral progeny release in cardiomyocytes, and dampened CVB-induced excessive T-cell proliferation that results in myocardial injury in a nitric-oxide (NO) dependent manner [74].

In a mouse model of acute HBV infection [75], the adoptive transfer of BM-MSCs ameliorated liver injury and decreased inflammation. However, the therapy also paradoxically increased viral replication, hypothesized to be partly due to MSC suppression of NK-mediated cell cytotoxicity. NK cells play a crucial role in viral clearance during acute HBV infection. This study did not explore MSCs’ effect on CD8 + T-cell function and focused mainly on short-term outcome without observing long-term progression of HBV infection post-MSC therapy.

In additional to cell-based approach, MSC-derived extracellular vesicles (EVs) may have comparable efficacy to MSC administration in a swine model of influenza. Khatri et. al found that the systemic administration of EVs isolated from swine BM-MSCs reduced nasal virus shedding and viral replication in lung tissue. The administration of EVs also altered pro-inflammatory mediator secretion and reduced histopathological evidence of injury when administered after viral inoculation in a mixed swine (H3N2, H1N1) and avian (H9N5, H7N2) influenza-induced pig lung injury model. The authors hypothesized that there was RNA transfer from EVs to epithelial cells [76]. These findings suggest systemic EV administration as a potential cell-free strategy for use in respiratory virus-induced lung conditions.

In our recent unpublished work, we have determined that feline adipose-derived MSCs were able to enhance granzyme B expression in CD8 + T-cells, shift their phenotype towards terminally differentiated effector cells (CD57 + , CD45RA + and CD62L-) and augment the ability of these cells to lyse virally-infected target cells in vitro [article under review]. Preliminary in vivo data also suggest that in feline chronic gingivostomatitis (FCGS), a disease associated with feline calicivirus infection, a positive response to MSC therapy also results in FCV clearance aligned with improvement in clinical disease [77].

Similarly, in an unpublished study of SIV infection model of AIDS in rhesus macaques, our group has demonstrated that MSCs enhance viral clearance by augmenting CD8 + T-cell and B cell activity through granzyme B upregulation and increased anti-SIV antibody production, respectively. In this model, MSCs enhanced viral particle transport to intestinal lymphoid follicles, and the development of robust germinal centers via Tfh induction which lead to enhanced viral clearance. Moreover, MSC treatment of SIV + rhesus macaques induced proliferation of CD8 + T-cells, in contrary to the suppression normally observed with MSC therapy [article under review].

Collectively, these studies in animal models suggest that MSCs have the ability to adapt their interactions with immune cell subsets in viral diseases in ways that are distinct from MSC interaction with immune cells when administered for diseases driven by immune-pathology mechanisms which aim to enhance the regulatory arms of the immune system.

## MSC Therapy in Human Clinical Trials of Viral Diseases

Upper until the time of article submission, there are currently 19 clinical studies registered involving the use of MSCs and/or their secretome to treat viral infection or the conditions associated with viral infection (ClinicalTrials.gov, Table 3). The most common therapeutic target (12/19; 63.2%) was for the treatment of respiratory problems associated with the novel Coronavirus infection (SARS-CoV-2; Covid-19). These trials are predominantly conducted with umbilical-cord derived MSCs (UC-MSCs). UC-MSCs are desirable for the treatment of acute viral infections due to their rapid doubling time in culture compared to BM-MSCs or AD-MSCs [78]. Robust MSC expansion facilitates the rapid generation of a therapeutic MSC dose in critically ill patients [79].

**Table 3:** Current clinical trials on the treatment of viral-associated diseases with multipotent stromal cells and/or their products

	Study Title	Treatment	Conditions	Status	Location
1	Treatment with Human Umbilical Cord-derived Multipotent stromal cells for Severe Corona Virus Disease 2019 (COVID-19)	UC-MSCs	Corona Virus Disease 2019 (COVID-19)	Completed	China
2	Efficacy and Safety of Umbilical Cord Multipotent stromal cells for the Treatment of Severe Viral Pneumonia	UC-MSCs	Corona Virus Disease 2019 (COVID-19)	Not yet recruiting	China
3	Umbilical Cord(UC)-Derived Multipotent stromal cells(MSCs) Treatment for the 2019-novel Coronavirus (nCOV) Pneumonia	UC MSCs	Corona Virus Disease 2019 (COVID-19)	Recruiting	China
4	Study of Human Umbilical Cord Multipotent stromal cells in the Treatment of Severe COVID-19	UC-MSCs	Corona Virus Disease 2019 (COVID-19)	Not yet recruiting	China
5	Bone Marrow-Derived Mesenchymal Stem Cell Treatment for Severe Patients With Coronavirus Disease 2019 (COVID-19)	BM-MSCs	Corona Virus Disease 2019 (COVID-19)	Not yet recruiting	China
6	A Pilot Clinical Study on Inhalation of Multipotent stromal cells Exosomes Treating Severe Novel Coronavirus Pneumonia	MSC-derived exosomes	Corona Virus Disease 2019 (COVID-19)	Not yet recruiting	China
7	Use of UC-MSCs for COVID-19 Patients	UC-MSCs	Corona Virus Disease 2019 (COVID-19)	Completed	USA
8	Umbilical Cord Tissue (UC) Derived Mesenchymal Stem Cells (MSCs) Versus Placebo to Treat Acute Pulmonary Inflammation Due to COVID-19	UC-MSCs	Corona Virus Disease 2019 (COVID-19)	Not yet recruiting	USA
9	Regenerative Medicine for COVID-19 and Flu-Elicited ARDS Using Longeveron Mesenchymal Stem Cells (LMSCs)	MSCs (unspecified source)	Corona Virus Disease 2019 (COVID-19)	Recruiting	USA
10	Clinical Use of Stem Cells for the Treatment of Covid-19	MSCs (unspecified source)	Corona Virus Disease 2019 (COVID-19)	Recruiting	Turkey
11	Treatment of Covid-19 Associated Pneumonia with Allogenic Pooled Olfactory Mucosa-derived Multipotent stromal cells	OM-MSCs	Corona Virus Disease 2019 (COVID-19)	Enrolling by invitation	Belarus
12	Therapeutic Study to Evaluate the Safety and Efficacy of DW-MSC in COVID-19 Patients	MSCs (unspecified source)	Corona Virus Disease 2019 (COVID-19)	Completed	Indonesia
13	Therapeutic Effects of Liver Failure Patients Caused by Chronic Hepatitis B After Autologous MSCs Transplantation	BM-MSCs	Liver Failure from HBV infection	Completed	China
14	Allogeneic Bone Marrow Multipotent stromal cells Transplantation in Patients with Liver Failure Caused by Hepatitis B Virus (HBV)	BM-MSCs	Liver Failure from HBV infection	Unknown	China
15	Clinical Study of Human Umbilical Cord Multipotent stromal cells(19#iSCLife®-LC) in the Treatment of Decompensated Hepatitis B Cirrhosis	UC-MSCs	Liver Cirrhosis from HBV infection	Recruiting	China
16	Umbilical Cord Mesenchymal Stem Cell for Liver Cirrhosis Patient Caused by Hepatitis B	UC-MSCs	Liver Cirrhosis from HBV infection	Recruiting	Indonesia
17	MSC for Treatment of CMV Infection	MSCs (unspecified source)	Cytomegalovirus infection after allogeneic hematopoietic stem cell transplantation	Unknown	China
18	Treatment With MSC in HIV-infected Patients With Controlled Viremia and Immunological Discordant Response	AD-MSCs	HIV	Completed	Spain
19	Umbilical Cord Mesenchymal Stem Cells for Immune Reconstitution in HIV-infected Patients	UC-MSCs	HIV	Unknown	China

Clinical trials using MSC therapy for Hepatitis B virus (HBV) infection and its associated liver disease are also reported (4/19; 21.1%). HBV infection is extremely widespread with over 350 million carriers around the world. HBV infection can result in hepatitis cirrhosis and hepatocellular carcinoma and there are limited treatment options available [80]. In a randomized controlled trial for HBV–related acute‐on‐chronic liver failure, infusion of allogeneic BM-MSCs significantly increased survival rate by improving liver function and decreasing the incidence of severe concurrent infections [81]. Based on a meta-analysis of MSC-based clinical trials for liver diseases, mechanisms involved in the efficacy of MSC therapy in HBV are focused on the hepatic reparative effects and/or restoration of T-reg/Th17 balance rather than on viral clearance [82]. With that said, concurrent in vitro work showed that BM-MSCs inhibited the expression of HBV DNA and enhanced viral clearance in HBV-infected lymphocytes [83]. Since BM-MSCs permit HBV infection, they may become reservoir of viruses after administration. However, AD-MSCs were found to be not susceptible to HBV [84] and may be a more suitable source of HBV-associated liver condition. Further studies on the long-term effects of MSC therapy on HBV infected individuals, from varying sources of MSCs, are necessary to determine its safety and efficacy in therapeutic use.

Clinical trials have also been conducted in patients with CMV and human immunodeficiency virus (HIV) infection. CMV is common, but infection is often asymptomatic except for in immunocompromised patients. CMV also remains a common complication after hematopoietic stem cell transplantation [85]. Paradoxically, CMV can infect and compromise MSC functions [86]. MSC therapy in the context of CMV may not be truly efficient and should be reserve for refractory cases as a further down option.

MSC therapy is also being applied to HIV infection as an adjunct for immunomodulation. For many patients, highly effective anti-retroviral therapy (HAART) can suppress circulating viral load and can increase life expectancy. However, some infected individuals are classified as nonimmune responders (NIR) and remain susceptible to opportunistic infections due to their low numbers of CD4 + T-cells. An in vitro study using latent HIV-infected cell lines reported a novel role for MSCs and MSC secretome in HIV-1 latency-reactivation through phosphoinositide 3-kinase (PI3K) and nuclear factor kappa-B (NFκB) signaling pathways [87]. A clinical trial published by Zhang et. al showed that UC-MSC therapy can increase the number of circulating naive and central memory CD4 + T-cells and restore HIV-specific IFN-γ and IL-2 production, evidence of systemic immune reactivation post-treatment and did not lead to increased viral loads [88]. However, in a early phase clinical trial for NIR, MSC infusions were found to not effectly improve immune recovery or reduce immune overactivation [89]. Supplemental in vivo research is needed to elucidate the effects of MSCs in reactivation of HIV-1 in host microenvironment.

## MSCs for the Treatment of COVID-19

**Fig. 3:**
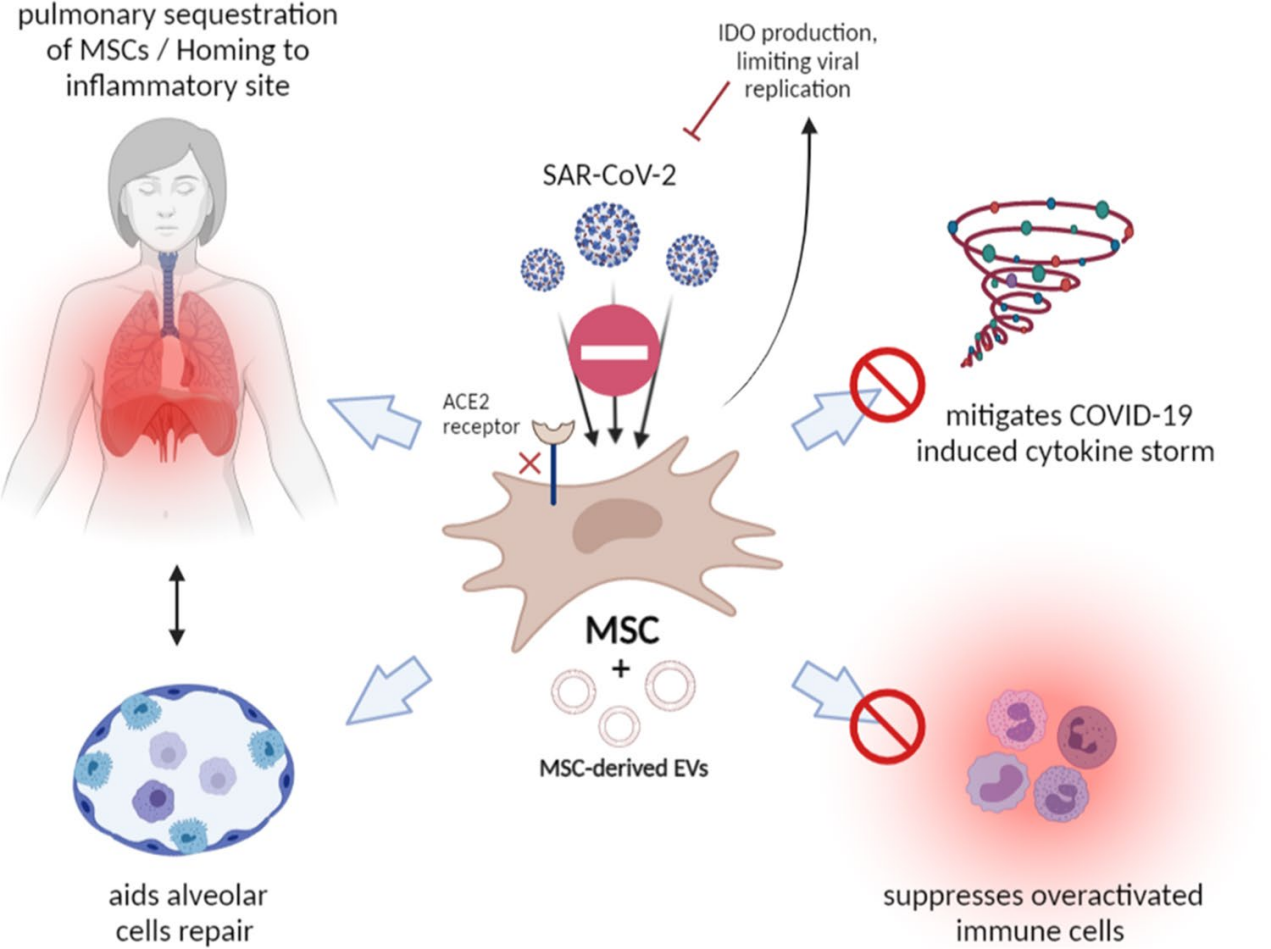
Potential use of MSC for the treatment of COVID-19

COVID-19, a newly-recognized infectious disease with rapid transmission of severe acute respiratory syndrome coronavirus 2 (SAR-SCoV-2) and has become a major concern all over the world. During the rush of finding novel treatment for the COVID-19 pandemic in addition to the traditional corsticosteroid therapy, covalescent plasma and neutralizing antibody cocktails [90–92], MSCs and their secreted products were explored as viable options due to their antiviral, anti-inflammatory and tissue regenerative capabilities (Fig. 3).

MSCs have been known to be sequestered in the lung after intravenous administration, creating a benefit in their utilization for the treatment of pulmonary disease [93]. Moreover, MSC-derived extracellular vesicles have shown to ameliorate inflammatory lung diease, including respiratory distress syndrome (ARDS), acute lung injury (ALI) and chronic obstructive pulmonary disease (COPD), from both infectous and non-infectious causes in several preclinical models [94]. In a completed clinical trial involving acute respiratory distress syndrome (ARDS) induced by epidemic influenza A (H7N9), MSC therapy significantly improved patients’ survival rate, lung function and decreased lung fibrosis [95].

Recent in vitro work [96] showed that despite their expression of angiotensin converting enzyme 2 (ACE2), a receptor for SAR-CoV-2 entry, human MSCs were resistant to their infection under steady-state, inflammatory condition and in the presence of SAR-CoV-2 infected cells. Moreover, SAR-CoV-2-exposed MSCs also retained their ability to secrete IDO [96], a mediator that can limit emergent viral biosynthesis through tryptorphan depletion pathway [97].

MSC therapy was implemented toward SAR-CoV-2 infection-induced pneumonia for the first time by Leng et. al in January, 2020 in 7 Covid-19 patients with promising outcomes. MSC administration was associated with an increase in the peripheral blood lymphocyte count and a concurrent decrease in C-reactive protein and activated cytokine-secreting immune cells, such as CXCR3 + CD4 + /CD8 + T cells and CXCR3 + NK cells [98]. Additionally, these Covid-19 patients became SAR-CoV-2 virus negative through RT-PCR detection 2 weeks after MSC administration [98]. According to the first published study, MSCs cannot be infected by SARS-CoV-2 and are deemed safe and effective in critical patients. The limitation of the aforementioned first study includes the lack of sufficient control group, small sample size and enrolled participants with only 1 patient in critical condition. However, several recent clinical trials and case reports reaffirm the beneficial use of MSCs in COVID-19.

In a few recent double-blinded, phase-2 randomized control trials utilizing UC-MSCs for acute respiratory distress syndrome, MSCs therapy significantly improved survival, reduced inflammatory cytokines and alleviated COVID-19 induced lung damage [99, 100]. The clinical trial conducted by Langzoni et. al also measured mean viral load of SAR-CoV-2 through qRT-PCR on day 0 and day 6 of treatment which did not differ between treatment and control group. However, it is interesting to note that some of the patients in their control group remained SAR-CoV-2 positive while all of the participants in group receiving UC-MSCs were negative [99]. Further kinetic study of viral clearance may be befinicial, given that viral load of SAR-CoV-2 has been shown marked correlation to the severity of acute respiratory distress syndrome [101].

Another trial using UC-MSCs and placental MSCs showed that COVID-19 patients with low white cell count and low lymphocyte count prior to therapy resulted in poorer outcomes [102], suggesting that aside from reduction of inflammatory responses through secreted paracrine factors, MSCs’ interaction with existing immune cells play an important role in successful treatment.

Overall, present data demonstrated in short-term studies that MSCs have shown efficacy in managing COVID-19 patient conditions with no obvious adverse effects. Pre-existing condition and other co-morbidities which may affect the potency of MSC therapy requires further investigation and confirmation.

## Conclusion

MSC therapy remains an option for the treatment of virus-associated diseases, especially those with sustained inflammation or those that require immunomodulation of skewed immune cell subsets. However, with certain virus infections, MSC therapy may result in enhanced viral replication, particularly in those in which MSCs are highly permissive to infection. In these cases, a cell-free therapeutic approach using EVs-derived from MSCs might be a promising approach to circumvent this issue.

Through TLR signaling and crosstalk between MSCs and effector immune cells, MSCs may maintain a crucial balance between enhancement of pathogen clearance and suppression of an overactive response. This interaction may help to preserve host cell integrity and facilitate tissue repair.

MSCs may react differentially under varying viral-associated conditions. It’s crucial to bridge the translational gap between the fundamental research of MSCs and their therapeutic applications. Studies that directly evaluate the ability of MSCs to clear virus are still limited. More robust animal models of viral diseases with larger sample size and well-designed randomized controlled clinical trials are needed to adequately assess their safety and potential of exerting antiviral effects on various viruses.

## Data Availability

Not applicable
